# Chicago Medical Society—Annual Meeting

**Published:** 1882-05

**Authors:** 


### Society Reports.

### ARTICLE VI.

CHICAGO MEDICAL SOCIETY. Annual Meeting.

The Chicago Medical Society held its annual business meeting at the Grand Pacific Hotel, on the evening of April 10, 1882, E. Ingals, M.D., President, in the Chair. After the minutes of the last meeting were read and approved, Dr. James G. Kiernan was admitted to membership, and Dr. B. Rel VanDoozer, graduate of Chicago Medical College, 1868, was proposed for membership by Drs. L. H. Montgomery and Wm.E. Quine.

Also, Drs. N. N. Hurst, graduate of Jefferson Medical College, 1872, and W. C. Maull, graduate of the University of Louisville, 1869, were proposed by Drs. G. O. Taylor and E. W. Sawyer.

The Secretary, Liston H. Montgomery, then proceeded to read his annual report, as follows:

*Mr. President and Members of the Chicago Medical Society:* —I have the honor to present the second annual report of the present incumbent as secretary of this society, which, summarized in brief, is as follows:

During the year 18 meetings have been held, and 304 members have been in attendance at 17 of the meetings, which does not include the one to-night. There were also 99 visiting physicians; or in all, 403 persons. Compared with the number in attendance the year previous, there has been an increase of 26 members and 25 visitors; or 51 persons. The average number of members in attendance at each meeting has been about 18; of visitors 6; or a total number of 24 physicians. Eighteen members joined the society, compared to 10 who were admitted last year.

At the last annual meeting 186 names comprised the roll of membership. Adding to this nnmber the 18 new members admitted, we have 204. This number has been decreased, however, as follows: Deaths, 3; resignation, 1; removal from the city, 3; and for obvious reasons 6 names have ceased to appear on the membership list. This deficiency of 13, then, leaves a corrected list of 191, and no less than this number of postal card announcements have been issued for each meeting. The following comprise the list of papers that have been read, many of which are of a high degree of merit, and as such are recorded in the minutes of the society:

(1) "Vaccination, and Varieties of Virus now in Popular Use."—Oscar C. DeWolf.

(2) "Rotheln, or German Measles."—Chas. W. Earle.

(3) "Numbers and Measures, with Scientific Corrections, with Reference to Dispensing Medicine."—F. M. Weller.

(4) "Report of a Case of Maternal Mother's Marks."—H. D. Valin.

(5) "Cremation."—Chas. W. Purdy.

(6) "Some Impressions of the International Medical Congress of 1881; being Personal Observations on the Section on Gynæcology and Obstetrics.'"—DeLaskie Miller.

(7) "Is there a Chronic Carbolic Acid Poisoning?"—Edmund Andrews.

(8) "Model of an Astygmatic Eye, demonstrating the Refractive Condition of the Organ."—Lytton Forbes.

(9) "Report of Cases in Ophthalmology."—E. L. Holmes.

(10) "Operation for the Total Extirpation of the Uterus (through the Vagina) with Recovery."—Chr. Fenger.

(11) "Predisposing Causes of the Alcoholic and Opium Habits."—Mary H. Thompson.

(12) "Disposal of the Dead."—W. H. Curtis.

(13) "Report of a Case where a Large Splinter of Wood had been Buried in the Superior Maxillary and Zygomatic Fossa for Fifteen Months, with Operation for Removal and Recovery."—Roswell Park.

(14) "Malarial Keratitis."—F. C. Hotz.

(15) "Report of a Case of Tetanus caused by a Foreign Body in the Orbit."—F. C. Hotz.

(16) Management of Parturition."—Ephraim Ingals.

(17) " Opening and Drainage of the Large Joints in Suppurative Synovitis."—Chr. Fenger and E. W. Lee.

(18) " Health Resorts."—Chas. E. Davis.

(19) A Report on the Treatment of Fifteen Cases of Typhoid Fever.—H. D. Valin.

(20) Fracture of the Frontal Bone, resulting in Necrosis of the Cranium."—John Bartlett.

(21) Supra-Malleolar Osteotomy."—Chr. Fenger and E. W. Lee.

(22) " Simulation of Insanity by the Insane."—James. G. Kiernan.

But few special committees has it been found necessary for the President to appoint, hence work in this direction has been limited during the past year. Especial mention is due, however, to the memorial committee appointed a few weeks since, who prepared a biographical sketch, with appropriate resolutions, of a deceased member. And the same compliment will apply to the committee which subsequently presented the "series of resolutions" to a near relative of the deceased.

To recapitulate, then, in a word: One more meeting has been held during the past year, and two more papers presented, than has been done before in some years, to our knowledge. The membership has been actively increased, and the Chicago Medical stands as the banner medical society of the city, the Illinois State Society excelling it only in numbers, not in talent, as many of our members belong to that body, as well as to the American Medical Association. During the past year, we were represented at the International Medical Congress that was held in London, and our delegates were received in medical and surgical associations in many other of the great European cities besides. At home, we were represented at the meeting of the American Public Health Association held at Atlanta during the latter part of the year 1881, and thus, has our influence been extended over a pretty broad expanse.

Before closing this report, your attention is invited to dwell upon two or three suggestions, which, if carried out, would, I trust, aid my successor in performing and lessening the duties of the secretaryship very considerably, and aside from this, as a member of the society. Our business would be transacted in a more general, professional manner, if we held our meetings once a month (say on the evening of every third Monday), and to do so the entire year around, for it is apparent to each of you, that the attendance after the vacation of the summer months, does not regain itself to that point which was previously the case before adjournment. Second. With the initiation of every new member, I would favor the plan of their being required to read, within three months from the time of their admission, an "inaugural thesis," as much, indeed, as annual dues should be exacted. To my mind, one or the other of the above methods (perhaps the latter plan) is an essential one, and an important step in the right direction.

In the discussions, I am sure none during the past year could have felt more at ease, for our worthy President did his part to have us all know that formality was not the popular method of discussing topics, and on a number of occasions, it was especially desired that practical suggestions should be advanced by new members in participating, who otherwise would not have done so, had not their views been solicited.

History informs us that this is our thirty-second year of existence, and our annual meeting one year hence will constitute the ter-centennial anniversary. Let us emulate somebody, if we cannot do it otherwise, and inaugurate a graceful custom of celebrating this ter-centennial anniversary of the society by having a splendid oration delivered (one gotten up expressly); and, as banquets seem to hold a high plane on the surface of æstheticism, close the business and exercises of the annual meeting with a fine banquet, etc., etc.

And now, before retiring from the distinction of secretary of this society, I wish to thank the members for courtesies extended, and my only regret is that I have not served you better, and that greater triumphs and achievements have not been the result; but which, I hope, are within the near future.

All of which is respectfully submitted by

LISTON H. MONTGOMERY, M.D.

Dr. C. T. Fenn moved that the secretary's annual report, just read, be accepted and adopted; also that a committee of three be appointed by the chair, to take action on the recommendations embodied in the report. The chair then appointed Drs. A. R. Jackson, R. G. Bogue, D. W. Graham, J. H. Hollister, committee. The chairman was appointed on the motion of W. H. Curtis, thus making the committee five in number, instead of three, as the original motion called for.

The treasurer, Dr. E. F. Ingals, presented his report, which is as follows:

RECEIPTS.

| | | |
|---|---|---|
| April 4, 1881, cash on hand | $156.32 | |
| April 4, 1881 to April 10, 1882, collections of annual dues and initiation fees | 323.00 | |
| | | $479.32 |

EXPENDITURES.

| | | |
|---|---|---|
| April 7, 1881, paid J. B. Drake & Co. for use of rooms | $50.00 | |
| April 4, 1881, to April 10, 1882, stationery, postage and writing | 15.35 | |
| April 4, 1881, to April 10, 1882, printing and postals for notices of meetings | 61.00 | |
| Paid collector commissions on delinquents | 6.90 | |
| | | $133.25 |
| Leaving balance on hand | | $346.07 |

The financial report was, upon motion duly seconded, adopted. A list of names was then read by the treasurer who were indebted to the society for two or more years' annual dues. Upon motion of Dr. L. C. Waters, that these members be expelled for non-payment came near being carried, when it was modified by carrying out article IV of the by-laws, which reads that their membership in the society is declared forfeited.

The election of officers was then proceeded with. Dr. G. C. Paoli moved, duly seconded, that the first ballot for President be informal. The following names, with the number of votes, was the first result:

Whole number of votes cast for President was 32—A. R. Jackson, 5; J. H. Hollister, 9; D. W. Graham, 6; E. Ingals, 4; DeLaskie Miller, 3; F. C Hotz, 3; G. C. Paoli, 1. Drs. Miller, Graham, Jackson and Ingals declined with thanks, when Dr. J. H. Hollister was chosen unanimously on the first formal ballot as President.

First ballot for Vice-President resulted as follows:

Whole number of votes cast, 33—D. W. Graham, 7; Emma Gaston, 15; A. H. Foster, 5; DeLaskie Miller, 2; S. J. Jones, 1; M. H. Thompson, 1; R. G. Bogue, 1; L. C. Waters, 1. Dr. Emma Gaston declined with thanks, and the second ballot resulted in Dr. D. W. Graham's receiving the majority, and he was declared elected Vice-President.

Firt formal ballot for Secretary was as follows:

Whole number of votes polled, 33—L. H. Montgomery, 20; C. W. Purdy, 1; A. P. Gilmore, 1; E. J. Gardiner, 6; L. C. Waters, 1; H. D. Valin, 1; A. H. Foster, 3. When the result was announced, Dr. L. H. Montgomery was declared elected to the secretaryship.

Upon motion of one of the members, the Secretary was authorized to cast the vote of the society for Dr. E. Fletcher Ingals, as Treasurer, which was accordingly done.

The new President was invited to take the chair, when the retiring President, Dr. E. Ingals, spoke as follows:

"I am glad of the opportunity to thank the members of the society for having, by their acts during the year, made the duties of its officers pleasant and easy. The history of the society, both in numbers and influence, was never before as good as it is to-day, and this will be advanced by every recurring year. When the last of our circle is gone, I doubt not this society will remain, and the labor we each individually give it will become a part of its history, and the influence we exert upon it may be so incorporated into its structure as to survive after our very names may be forgotten. But though we have done well, we have done less than we ought. Every one worthy the name of a physician owes much to his profession. All scientific organizations which have its advancement for their object should receive their hearty support, and especially should our medical teachers contribute generously to their transactions. It is true, many individual engagements demand their attention, but they should not neglect society work. Where much is given, much should be required. By a more thorough performance of their duties in this matter, they would greatly benefit the profession, and in return they would be benefited themselves—possibly in a still greater degree, for it is the law of all labor that the greatest reward is to him who does the work, for the reason that such work strengthens and improves the mind and builds up the character, and these results have worth above and beyond all that is external and transitory.

"Chicago is already among the important medical centers of this country, and we should each of us aid as much as we may to give it a more excellent rank. The names of the best known medical men of this nation constantly appear in the transactions of the various scientific associations of the large Eastern cities, and the credit they are to the profession is reflected upon themselves.

"Chicago is outgrowing its childhood, and it should make its manhood known by its works. That it will do this I have a firm assurance, and let us all, individually and collectively, help to push forward the good work."

A motion by Dr. F. M. Wilder was duly seconded, that the thanks of the society were due the retiring President, and all the other officers, and should be spread upon the records, for their services. Carried.

Dr. E. Ingals then moved that $50 be donated to Mr. John B. Drake for the use of the parlors the past year—also that a token of regard in the form of $25 be voted the Secretary. Both motions were carried unanimously.

Dr. D. W. Graham, on behalf of the memorial committee appointed a few weeks ago, offered the following report on necrology:

Dr. James Allen Mead was born in Otsego county, New York, February 8, 1841. He died in this city February 16, 1882, after an illness of two weeks, of typhoid fever. He received a common-school education in his native place, and took an academical course at Cazenovia, N. Y.

Eighteen years ago he came to Chicago, and engaged in the retail drug business. He studied medicine in Rush Medical College, and graduated in 1876. The last two years of his life were spent exclusively in the practice of medicine, in which he had a good measure of success. He became a member of this society in 1881. Your committee, in addition to the foregoing history, would respectfully offer the following resolution, as expressing the sense of this society, and ask that it be spread upon the records:

That in the death of Dr. Mead, this society, and the medical profession, have lost a worthy member, whose private and professional life were characterized by the highest honor and integrity.

JOHN BARTLETT,<br>

G. C. PAOLI,<br>

R. C. HAMILL,<br>

D. W GRAHAM, } Committee.

The committee on membership and miscellaneous business, consisting of Drs. G. C. Paoli, J. H. Etheridge and E. L. Holmes, was continued.

The hour was deemed too late to hear the papers read, as announced, hence a motion to defer their reading until next meeting was carried.

The society then adjourned.

LISTON H. MONTGOMERY, *Secretary.*

---

### ARTICLE VII.

CHICAGO MEDICAL SOCIETY.

